# A Systematic Review and Meta-Analysis of Associated Factors of Gender-Based Violence against Women in Sub-Saharan Africa

**DOI:** 10.3390/ijerph18094407

**Published:** 2021-04-21

**Authors:** Muluken Dessalegn Muluneh, Lyn Francis, Kingsley Agho, Virginia Stulz

**Affiliations:** 1 School of Nursing and Midwifery, Parramatta South Campus, Western Sydney University, Sydney, NSW 2151, Australia; L.Francis@westernsydney.edu.au; 2 Amref Health Africa in Ethiopia, Addis Ababa P.O. Box 20855, Ethiopia; 3 School of Science and Health, Western Sydney University, Locked Bag 1797, Perth, NSW 2571, Australia; K.Agho@westernsydney.edu.au; 4 African Vision Research Institute (AVRI), University of KwaZulu-Natal, Durban 4041, South Africa; 5 School of Nursing and Midwifery, Western Sydney University, Locked Bag 1797, Penrith, NSW 2751, Australia; v.stulz@westernsydney.edu.au

**Keywords:** gender-based violence, risk factors, ecological, sub-Saharan Africa, cross-sectional, systematic and meta-analysis

## Abstract

A systematic review and meta-analysis were employed to address the associated factors of gender-based violence (GBV) in sub-Saharan African (SSA) countries. The Preferred Reporting Items for Systematic reviews and Meta-Analysis guidelines were followed. Ovid Medline, CINAHL, Cochrane Central, Embase, Scopus and Web of Science were used to source articles with stringent eligibility criteria. A total of 4931 studies were found and 50 studies met the inclusion criteria. Pooled meta-analyses revealed that low educational attainment, higher alcohol consumption, substance use, history of child and family abuse, limited decision-making skills, experiencing depression, males having multiple sexual partners, and younger age were found to be individual- and family-associated factors that increase the experiences of GBV. Community tolerant attitudes to violence, women’s unemployment, being Muslim, lower socioeconomic class, food and social insecurity were found to be community- and societal-associated factors of GBV. Alcohol consumption, low educational attainment, experiencing depression, being younger, a history of child and family abuse, tolerant attitudes to violence, and low socioeconomic status were poignant factors associated with GBV amongst women in SSA countries. The need to develop a multipronged approach of intervention is a top priority in SSA to reach the Sustainable Development Goals’ (SDGs) target of 2030 to eliminate all forms of violence. Socio-behavioural change communication interventions at individual and community levels need to be introduced, and interventions need to address the prevention of child and family abuse and increase women’s feelings of empowerment in order to prevent GBV in SSA.

## 1. Introduction

Globally, GBV is an important public health problem and human right violation challenge, worsening in developing countries including SSA [1,2]. The experience of GBV threatens the lives of many women and limits the autonomy of women to address health as a primary concern for themselves and their children [3]. According to the United Nations (UN), GBV is defined as “any act of gender based violence that results in, or is likely to result in, physical, sexual, or mental harm or suffering to women, including threats of such acts, coercion or arbitrary deprivation of liberty, whether occurring in public or in private life” [1,3]. GBV encompasses physical, sexual and psychological abuse from intimate and non-intimate partners [1,3].

Worldwide, it is estimated that one in three women will experience at least one form of GBV during their lifetime experience, from as young as 15 years of age [1]. The prevalence of GBV is intensified in developing countries, such as SSA countries, where socioeconomic status is low and access to education is limited [4]. According to a systematic review and meta-analysis study conducted in SSA in 2020, the prevalence of IPV is as high as 44% and 14% for non-IPV [5], and is one of the worst regions for reporting GBV in comparison to global dimensions [4]. Researchers further explained that the prevalence rate might be higher if there were no barriers to reporting these cases. The barriers include: fear of stigma, revenge, women considering the matter as a private issue, fear of financial barriers and lack of law enforcement, just to mention a few [4].

This study only focused on SSA countries due to evidence showing GBV prevalence is high this region [5] and most available studies are limited to developed countries [1,3,4]. Moreover, researchers reported that factors related to GBV are complex and most available studies are restricted to developed countries, with limited evidence focused on SSA countries [1,2]. Some factors are protective against GBV and other factors may increase the likelihood of GBV [6]. Hence, understanding the factors related to GBV aims to contribute to the field of GBV and complement the existing evidence in order to design comprehensive interventions and inform policy makers.

Various theoretical approaches, views and frameworks have been adopted to explain the potential associated factors that might increase the chance of GBV including gender perspectives or feminist theories and sociological and ecological theories [7]. The ecological framework is the most widely used framework in public health research on GBV [3,7]. The ecological model considers the complex interplay between individual, relationship, community and societal factors that may lead to GBV [7]. This review applied the ecological framework as an exploratory tool to systematically explore associated factors of GBV.

## 2. Methods

The Preferred Reporting Items for Systematic reviews and Meta-Analysis (PRISMA) guidelines were followed [8]. PRISMA is an evidence-based minimum set of items for reporting in systematic reviews and meta-analyses. PRISMA focuses on the reporting of reviews, evaluating studies, and is used as a basis for reporting systematic reviews of various types of research, particularly evaluations of interventions. Ovid Medline, CINAHL, Cochrane Central, Embase, Scopus and Web of Science were used to source articles with stringent eligibility criteria. The search also included grey literature. CASP is a set of critical appraisal tools designed to be used when evaluating research. CASP has appraisal checklists designed for use with systematic reviews, randomised controlled trials, cohort studies, case–control studies, economic evaluations, diagnostic studies, qualitative studies and clinical prediction rules [9]. The quality of included studies was appraised using Critical Appraisal Skills Program (CASP) for cross-sectional studies [9]. The overall quality of each paper was rated on a ten-point scale that was scored from zero (none of the quality measures were met) to ten (all of the quality measures were met). The quality of the paper was based on the sum of points awarded, representing the overall quality score of the study. Studies were rated as poor quality (score ≤ 6), medium quality (7–8), and high quality (≥9) (Table S1). There were no studies excluded due to poor quality. Data were extracted from studies using a specially developed Excel tool for data extraction.

Two reviewers independently extracted and assessed the quality of the articles. Studies published in SSA countries between 2008 and 2019 were included. The year 2008 was used as a baseline as a year of a greater global commitment to addressing violence against women; there has been a rapid expansion in the number of population studies examining GBV [10] since that time. Gender-based violence, intimate partner violence, domestic violence, physical abuse, emotional violence, reproductive coercion, sexual assault, sub-Saharan countries, women aged 15–49 years, determinants predictors and associated factors were the search terms and words used when performing the review search (Table S1).

Studies were included if they (i) focused on the associated factors of GBV; (ii) included females in the age range of 15–49 years; (iii) were studies conducted in SSA countries [11] including Benin, Burkina Faso, Cape Verde, Gambia, Ghana, Guinea, Guinea-Bissau, Ivory Coast, Liberia, Mali, Mauritania, Niger, Nigeria, Senegal, Sierra Leone, Togo, Cameroon, Central African Republic, Chad, Congo Republic-Brazzaville, Democratic Republic of Congo, Equatorial Guinea, Gabon, Sao Tome and Principe, Angola, Botswana, Lesotho, Mozambique, Namibia, South Africa, Swaziland, Zambia, Zimbabwe, Burundi, Comoros, Djibouti, Eritrea, Ethiopia, Kenya, Madagascar, Malawi, Mauritius, Rwanda, Seychelles, Somalia, Somaliland, Tanzania and Uganda; (iv) were published in English from 2008 to 2019; and (v) were quantitative studies

Gender-based violence was measured using the Demographic and Health survey (DHS) tool. The following key questions focused on experiences within women’s relationships, perpetrated by their husband/partner. Women were asked about physical violence experiences including push you, shake you, or throw something at you, slap you; twist arm or pull, punch you with his/her fist or with something that could hurt you; choke you or burn you on purpose; threaten or attack you with a knife and gun. Sexual violence experiences included: physically force to have sexual intercourse, physically force to perform any other sexual acts without need, and force you with threats or in any other way to perform sexual acts. Emotional violence experiences include say or do something to humiliate; threaten to hurt or harm you or someone close to you; and insult you or make you feel bad.

The metan command provides methods for the meta-analysis of studies with two groups. The effect measure with binary data is the difference between the ratio of two proportions (risk ratio), or the odds ratio. The syntax “metan” in Stata version 16.0 [12] was used to generate forest plots for each of the pooled effects. In a forest plot, the contribution of each study to the meta-analysis (its weight) is represented by the area of a box whose centre represents the size of the treatment effect estimated from that study (point estimate). A test of heterogeneity of each of the study datasets was obtained for the different authors and showed levels of inconsistency (I^2^ > 50%), thereby warranting the use of a random effects model in all the meta-analyses. The I^2^ statistic describes the percentage of variation across studies that is due to heterogeneity rather than chance. The metabias command performs the tests for funnel plot asymmetry proposed by Egger [13]. Sensitivity analyses were attended to examine the effect of outliers [13]. Egger’s test was used for publication bias. Publication bias is usually defined as the tendency for authors to publish studies with significant results. This bias might be for various reasons; for instance, selective publication of studies based on the direction and magnitude of their results, and studies without statistical significance (negative studies) being less likely to be published. Biased results from pooling the results from published studies alone are some of the examples.

## 3. Results

### 3.1. Description of Included Studies

**Figure 1:**
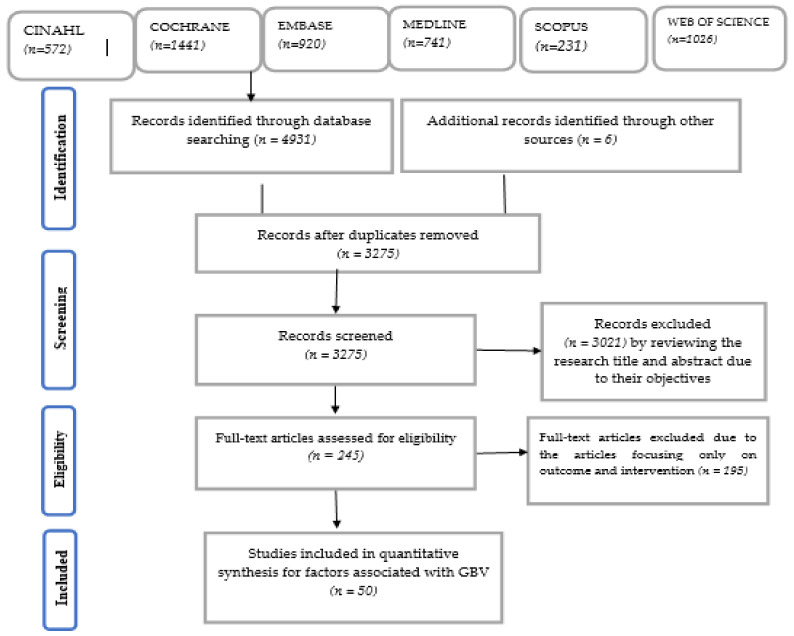
PRISMA flow chart that describes selection process of GBV articles that met inclusion criteria.

A total of 4931 articles, citations and 6 grey literature articles were included. After removing duplicates, 3275 titles and abstracts were screened. Of these, 245 full-text articles were retrieved. Fifty studies met the inclusion criteria about the associated factors of GBV (Figure 1).

Fifty articles met the inclusion criteria and were reviewed for data analysis and interpretation (Table 1). The majority of studies were cross-sectional studies; sample size ranged from 150 to 86,024, and random sampling techniques were used to attempt to reduce bias (Table S1).

### 3.2. Associated Factors of GBV 

This systematic review and meta-analysis report has been organised using the ecological framework that correlates individual, relationship, community and societal levels.

#### 3.2.1. Individual Level Risk Factors of GBV

Education status of women and partner- In this review, researchers found that women’s low levels of education attainment were associated with a higher number of GBV experiences [15,40,62,63]. Similarly, other researchers found that the educational level of the woman’s partner was an important factor for women experiencing GBV [6,22,31,40,56]. This review has highlighted that if both the woman and her partner have higher educational levels, they are less likely to experience GBV [31].

**Figure 2:**
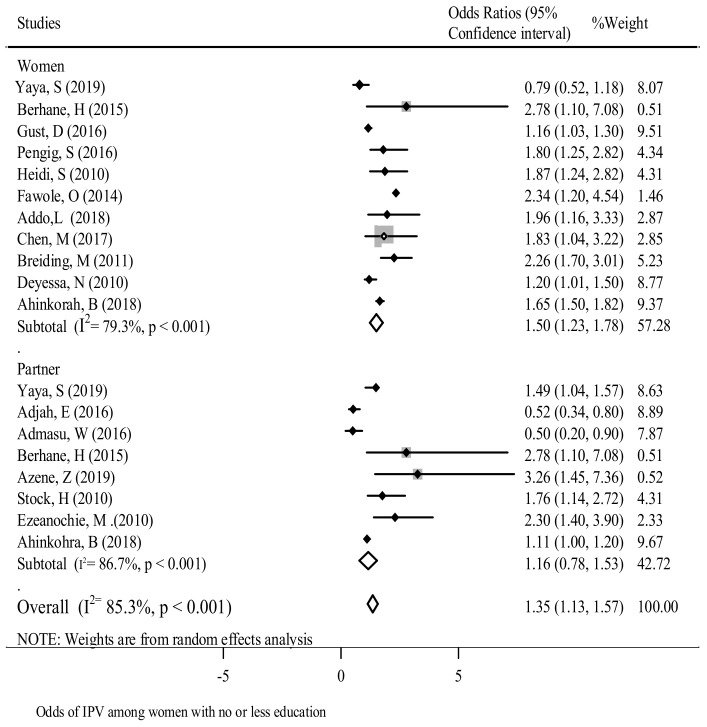
The association between education and experience of reporting GBV in SSA.

Overall, the meta-analysis showed that there was a significant association [OR: 1.35; 95% CI (1.13, 1.57)] between women and their partner’s educational level and experiences of GBV. Moreover, the subgroup analysis showed that the higher educational level of the woman was the most important factor [OR: 1.50; 95% CI (1.53, 1.78)] in reducing the chances of experiencing GBV (Figure 2). Egger’s test (*p* = 0.273) shows no publication bias (Figure S2). The I^2^ statistic describes the percentage of variation across studies that is due to heterogeneity rather than chance

Alcohol consumption - In various studies, alcohol consumption by women or their partner was found to be positively associated with GBV. Women who drank alcohol on a daily basis were more likely to experience GBV in comparison to women who did not drink alcohol [15,27,36,37,40,46,53]. Similarly, the likelihood of reporting GBV amongst women was very high when their partners consumed alcohol on a daily basis in comparison to women whose partners did not consume alcohol [16,21,40,48,49].

**Figure 3:**
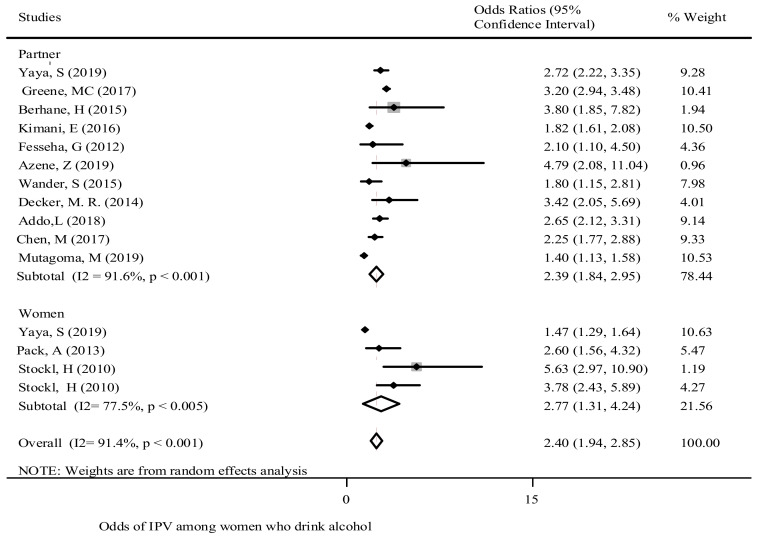
The association of drinking alcohol and experience of reporting GBV in SSA.

The pooled OR revealed that drinking alcohol increased the odds of women experiencing GBV by 2.4-fold [OR: 2.4; 95% CI (1.94, 2.85)]. The subgroup analysis showed the highest chances of violence occurred when women drank alcohol (Figure 3). The heterogeneity (Egger test *p* = 0.71) is shown in Figure S3. 

**Figure 4:**
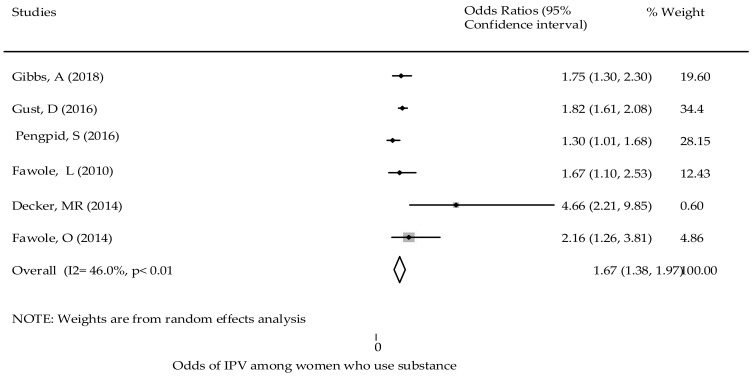
The association of substance use and the experience of reporting GBV in SSA.

Substance use - Substance use was one of the factors that was positively associated with women’s experiences of GBV [35,44]. A multi-country study showed that women who used marijuana over the course of their lifetime were 4.7 times more likely to experience GBV [OR: 4.66; 95% CI (2.21, 9.85)] in comparison to non-users. The pooled effect of substance use was significantly associated with women’s experience of GBV. Women using substances were twice as likely to experience GBV in comparison to those who did not [OR: 1.68; 95% CI (1.5, 1.89)] (Figure 4) with the Egger test (*p* = 0.551) (Figure S4).

Age of women and partners - In this review, many of the findings revealed that young women who were 15 to 19 years of age experienced a higher prevalence of GBV than adult women [6,14,31,46,54,63,64]. Two studies found that moderate age differences between partners were associated with high levels of GBV [49,54]. In contrast, other studies showed greater differences in age were less likely to experience IPV [15,49] (Table 1).

**Figure 5:**
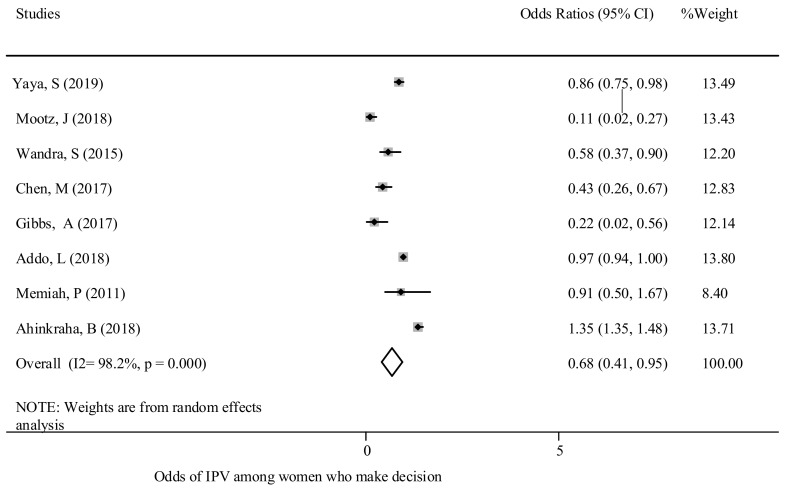
The association between GBV experience and decision-making power.

Decision-making skills - In this review, women’s autonomy to make a decision was an important predictor in whether she was likely to experience GBV. For instance, women were classified as participating in decision-making if they made decisions alone or jointly with their partner and included when women decided on major purchases, own health care access and visits to family. Women having strong decision-making skills were positively associated with lower levels of experience of GBV [15,29,45,47,50,65]. The overall pooled effects of decision-making skills with experiences of GBV were significant [OR: 0.68; 95% CI (0.41, 0.95)]. Women who have better decision-making skills had a 32% lower chance of experiencing GBV in comparison to those who had little decision-making skills (Figure 5). Egger’s test demonstrated no publication bias (*p* = 0.426) (Figure S5).

**Figure 6:**
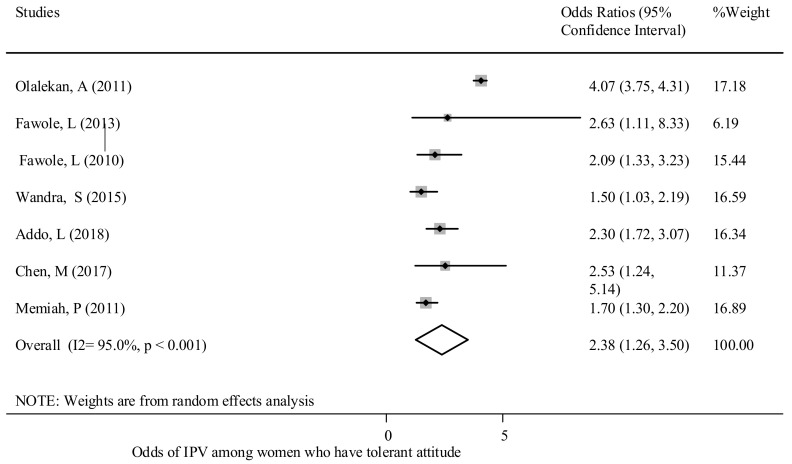
The association between tolerant attitude and experience of reporting GBV in SSA.

Attitudes of women and partners - This review found that experiences of GBV were associated with attitudes of women and their partners. Women and men who are more tolerant of GBV are more likely to consider abuse against women as being normal [17,37,47,48,49]. The overall pooled effect showed that women who were more tolerant towards GBV were twice as likely [OR: 2.38; 95% CI (1.26, 3.5] to experience GBV in comparison to those who were intolerant (Figure 6). Egger’s test (*p* = 0.033) results are shown in Figure S6.

Experiencing depression - This review found that GBV was positively associated with experiencing depression. In six studies, the findings showed that women experiencing depression or depressive symptoms were more likely to experience GBV [20,40,46,48,66]. For instance, a study conducted in SSA countries showed that women were almost two-thirds (63%) more likely to experience sexual violence if they were experiencing post-traumatic stress disorder (PTSD) [40]. A similar trend was reported in other studies [20,46].

**Figure 7:**
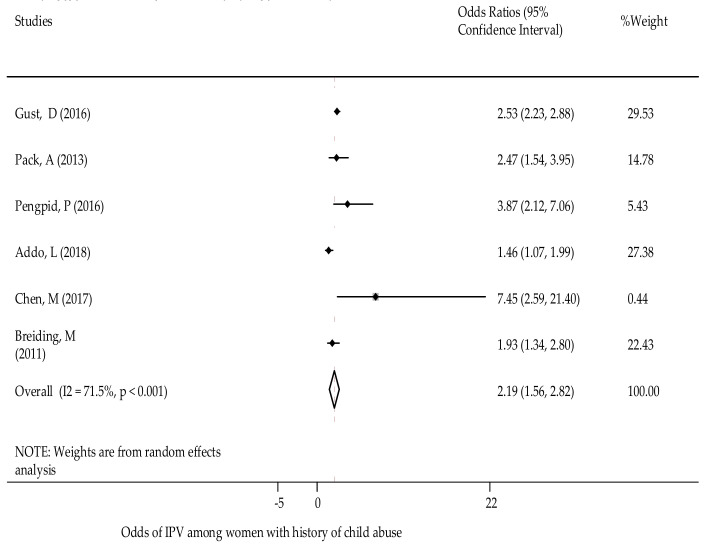
The association of history of child abuse and GBV experience.

History of child abuse - Researchers found that experiences of child abuse were positively associated with experiences of GBV against women [35,53,67]. The occurrence of GBV was found to be higher among women that had experienced physical, sexual and emotional abuse as a child [48,52,53]. Overall, experiencing child abuse was found to be a predictor of GBV in the later life span of women. Women who experienced child abuse were 2.3 times more likely to experience GBV in comparison to women who did not experience child abuse [OR: 2.33; 95% CI (1.71, 2.96)] (Figure 7). The test showed no publication bias (Egger’s test *p* = 0.61) (Figure S7).

#### 3.2.2. Relationship Risk Factors of GBV

Males having multiple sexual partners - The odds of reporting GBV was found to be higher amongst women whose partners had multiple sexual partners [23,35,36,46,48]. A study conducted in African countries indicated that males with two or more sexual partners over the past year increased the chance of women experiencing GBV by 1.78 times [35]. Similarly, a study in Nigeria showed polygamous unions doubled the chance of experiencing GBV [36].

**Figure 8:**
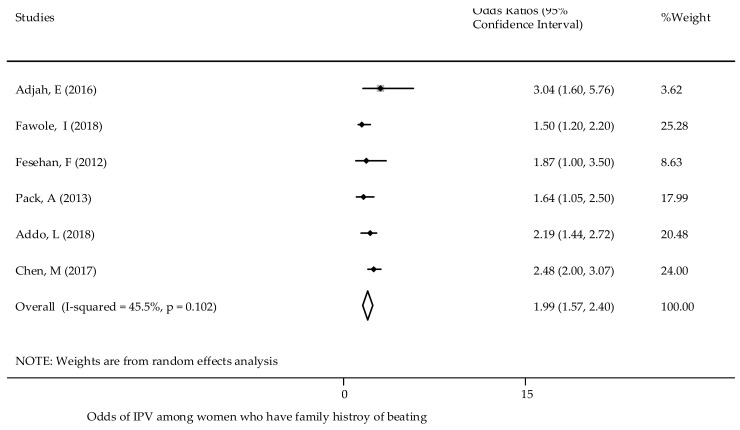
The association of family history of physical violence and experience of reporting GBV in SSA.

Previous history of parental abuse - A previous history of parental abuse (if women experienced abuse as a child by a parent) was positively associated with current experiences of GBV [21,48,68]. The chances of experiences of violence against women were higher if a woman had a parental history of violence [21,36] (Table 1). The overall pooled estimate found there was an association between a previous family history of violence and experiences of GBV. Women who had a parental history of violence were twice as likely to experience GBV (Figure 8). There was no publication bias (Egger’s test *p* = 0.715) (Figure S8).

Living arrangement with partner and early marriage - Studies have found that early marriage was associated with GBV [14,63]. The odds of GBV were higher if the woman was married, cohabiting, or having a relationship [26,31,54]. Similarly, studies showed women living without their partner were less likely to experience GBV as compared to women living with their partner [31,40] (Table 1). 

#### 3.2.3. Community Level Risk Factors of GBV

Community Attitude towards violence - Community attitudes towards GBV were a strong predictor for the occurrence of any form of GBV [49,60,67]. Women with more tolerant attitudes towards GBV in the community were 41% more likely to experience spousal violence [67]. Another study found that if the community accepted violence, women were four times more likely to experience IPV [49]. A study was conducted in Rwanda about health workers’ experience of workplace violence or any form of discrimination, including gender discrimination (defined as “any distinction, exclusion or restriction made on the basis of socially constructed gender roles and norms that prevents a person from enjoying full human rights”) [69]. Gender equality lowered the odds of health workers experiencing violence [60,69].

Employment and occupational status - Researchers found that employment increased the likelihood of experiencing or reporting GBV [50,58]. A multi-country study revealed the odds of experiencing IPV was 33% higher amongst women who were employed in comparison to those who were unemployed [58]. Researchers found that health care workers were more likely to experience GBV [57,58,61], particularly emotional violence. Nurses and midwives [OR: 4.06; 95% CI (1.20, 13.74)] were more likely to experience workplace violence in comparison to other health care workers [28,30] (Table 1).

Area of residence - Some studies found that women residing in rural areas were more likely to experience GBV in comparison to women in urban settings [31,34,63]. In contrast, a multi-country study indicated that women living in rural residences were 20% less likely to experience GBV in comparison to women living in urban residences [15,21,50].

#### 3.2.4. Societal Level Risk Factors 

Socioeconomic status - Nine studies reported the association between socioeconomic status and GBV. Most studies found that lower socioeconomic status was positively associated with experiencing GBV [15,16,49,50,58,62]. Researchers identified that women with a poor wealth index were more likely to report GBV in comparison to women with a higher wealth index [50,58] (Table 1).

Food and Social Security - Researchers found that poor food security had a positive association with violence against women. Two studies in South Africa found that household food insecurity was positively associated with women experiencing GBV [38,45]. A study conducted in Côte d’Ivoire [50] found that poor social security and lack of social freedom were associated with experiencing IPV. Additionally, this review showed that police refusing to protect women against violence was associated with physical [OR: 2.8; 95% CI (1.7, 4.4)] and sexual violence [OR: 3.0; 95% CI (1.9, 4.8)] [50].

## 4. Discussion

This comprehensive systematic review provides a plethora of information on the overall associated factors that augment the occurrences of GBV in SSA countries. This systematic review has identified that women’s experiences of GBV in SSA are associated with many factors that are related to individual, interpersonal, community and societal levels.

### 4.1. Individual and Relationship Factors

Poor education status, alcohol consumption, substance use, young age, limited decision-making skills, tolerant attitudes towards violence, experiencing depression, history of child abuse and parental abuse (if woman was abused by a parent), males having multiple sexual partners, living arrangements and early marriage were the most consistently identified individual and relationship risk factors that increased the experiences of GBV in SSA countries. Women’s alcohol consumption, tolerant attitudes towards violence and substance use were positively associated with experiencing GBV. This might be related to women’s inability to anticipate the potential risks, consequences and lack of conscious reflection towards GBV as a result of alcohol and substance use. Hence, women that do not consume alcohol or substances may reflect more positively about their circumstances in comparison to those who practise risky behaviours [47]. This is consistent with many other studies [10,50,64]. Women may be exposed to GBV from an early age and this might be related to the patriarchal situation of men’s dominance in SSA countries [24,70]. The following paragraphs will provide a more comprehensive discussion about these factors.

Secondary and higher educational status is the most important factor that will reduce experiences of GBV. Studies have shown that women who are better educated have a heightened understanding about preventing GBV as they are more conscious and aware of GBV in their community [15,36,71,72]. Higher education predisposes women to the effects of GBV in society and exposes women to global discourses and discussions that reject any form of violence. Women who are less educated may not understand how to implement mitigation measures against violence. They are also more likely to have limited exposure to legal support and herald tolerant attitudes to GBV. Women in SSA are mostly uneducated and, therefore, are likely to be subjected to the patriarchal male dominance that infiltrates their communities and increases the chances of being exposed to GBV. This situation poses other health and economical challenges.

Increased decision-making skills enable women to be empowered about understanding the availability of resources and this may contribute to varying power levels within the couple’s relationship that may influence more accessibility to health, social and economic services. As a result, if women are able to exercise choice and control within their relationship, they are less likely to experience GBV [73,74]. However, researchers suggest that developing shared decision-making skills will assist in preventing IPV. If women have high decision-making skills and are not inclusive of their spouse, they are more likely to experience GBV [19,29,58,65]. Decision-making skills are more compromised when the woman and her partner drink excessive alcohol and this may influence and increase any violence or criminal activities [3]. If women or their partners consume excessive amounts of alcohol, this may impair a person’s judgement and increase the likelihood of violence [19,29,65].

Women who experience depression were more likely to experience GBV. These findings are consistent with studies conducted in various countries [20,75]. GBV is more likely to occur in women with low self-esteem and women who fear humiliation, stigma and discrimination that further aggravate experiences of depression [2]. Women experiencing depression are more likely to roam the streets in SSA and are, therefore, more prone to experiencing GBV [20,75].

Women who have experienced previous child abuse are more likely to experience GBV as adult women. In this study, we found that previous child abuse doubles the chances of adult women experiencing GBV. This intergenerational cycle of violence might be attributed to continual exposure to violence during their childhood and increases the chances of women experiencing GBV later in their adult life. Children’s exposure to GBV poses long-term health and social consequences. This is strongly correlated with the sociological theory of violence. This means that violent acts within a family are reinforced during childhood and increases the chances of violence being experienced as adults. There is an assumption that there is an “intergenerational transmission” or “cycle of violence” [7]. This positive association has been described in many other studies [1,7].

This review has shown that partners’ promiscuous behaviour is an important predictor of experiencing GBV amongst women. The identified relational factors include alcohol consumption, having multiple sexual partners, previous history of abuse, and negative attitudes of partners, which significantly increased the occurrences of GBV. Consistent with these findings, many other developed countries and some African countries found an association between the partner’s promiscuous behaviour and experiences of GBV amongst women [9,63,72,73]

Although there is a tendency of reducing polygamy throughout SSA, men continue to exercise sexual freedom by having multiple sexual partners even whilst they are married or cohabitating with a woman. Having multiple sexual partners was found to be an important relationship risk factor for occurrences of GBV. This may be related to disrespect towards their female partners and may cause detachment, resulting in poor psychological and emotional bonding. The partner’s promiscuous behaviour may also expose women to sexually transmitted infections including HIV [4,73].

Similarly, the review revealed women are at higher risk of IPV if they marry before the age of 18 years. In many African countries, one of the drivers of early marriage is the family belief that if a young woman marries before having sexual intercourse, they will be protected from physical and sexual assaults, although this does not occur in reality in terms of IPV. There are a myriad of potential reasons why early marriage leads to higher levels of violence and this might be attributed to married women being less educated, being disempowered economically, and being influenced by her partner and the community, resulting in low decision-making skills and lack of autonomy. At the same time, greater age differences between partners are more likely to result in increased levels of GBV [14,34,63,64]

### 4.2. Community and Societal Factors

In this review, a limited number of factors were identified at the community and societal levels. This is mainly due to the limited research available about risk factors, rather than reflecting a true picture about experiences of GBV.

A community harbouring tolerant attitudes to violence was positively associated with higher levels of women experiencing GBV. Communities that harboured adverse attitudes towards violence were less likely to experience GBV. These communities were likely to support gender equities. Unfortunately, in most African countries, the system is patriarchal and prejudiced towards promoting male dominance, and fear was also a contributory factor of not reporting violence [1,2,16,67,69].

This review found that GBV was more likely to occur amongst employed women in comparison to unemployed women. This finding could reflect men’s disapproval of women’s independence and them being socially and financially stable. There could be a community expectation that a woman’s place is in the home as a family carer. Consequently, partners may be more likely to abuse women, especially if the partner is not the main breadwinner and is unemployed. The majority of employed workers in SSA are male and this predisposes the workplace towards more lenient laws against GBV. Women are also more likely to work in lower paid positions, increasing the chance of supervisors being perpetrators of violence. Alternatively, women who are unemployed are more likely to remain silent about experiencing violence due to fear of unrest within the family, harbouring tolerant attitudes towards violence and being economically dependent on their partner [2,24,67,70].

The socioeconomic status of women in the community and food security were found to be important societal predictors for experiencing GBV. Lower socioeconomic status and limited food security were generally associated with higher levels of experiences of GBV. Women may have limited access to different services, including educational services. Other reasons may be due to overcrowding of living areas and being dependent on others, which predisposes them to subordinate acts of violence [2,76]. Many women migrate to urban settings and engage in prostitution to earn their income and this increases the chance of GBV in many young African women. Studies showed that female sex workers are more likely to experience higher GBV in comparison to other women [1,5,74,77].

Furthermore, this review has highlighted that if both a woman and her partner have higher educational levels, resources or higher socioeconomic class, this is positively associated with lower levels of GBV. However, the level of violence was also associated with the partner’s individual risk factors. Gender inequalities could also account for consequences such as divorce and increased revenge including homicide, trafficking and stigmatising women, which is consistent with various studies [75,76].

### 4.3. Strengths and Limitations of This Review

This is the first systematic review and meta-analysis that has further explored SSA countries and will assist in designing an intervention. A rigorous search was conducted from multiple electronic databases. A quality assessment was conducted and two independent reviewers conducted the screening. Stringent eligibility criteria were developed. Despite this, the search only included articles published in English. All the studies were observational in design and only established relationships between variables, rather than causative factors. The quality of the evidence was dependent on the credibility of the original research findings. The study was not supported by qualitative studies.

### 4.4. Implications for Social Policy, Practice, and Research

This study provides vital evidence to inform policy and guide social and health organisations to respond and to prevent violence in alignment with the Sustainable Development Goals (SDGs) (also known as the Global goals and adopted by all United Nations Member States in 2015 as a universal call to action to end poverty, protect the planet and ensure that all people enjoy peace and prosperity by 2030). SDG 1 relates to no poverty and SDG 5 relates to gender equality [78]. Many factors were identified that are associated with increased experiences of GBV. Hence, prioritising the factors and the prevention of violence is an integral component of individual, human, social and economic development agendas that should commence sooner rather than later.

These research findings provide extensive evidence to develop integrated individual- and family-tailored interventions that address the prevention of GBV. This work should extend to the community to initiate social changes that will address gender inequity and economic development to improve the living conditions and socioeconomic status of women and their families. This research has provided further stimulus to develop interventions that target GBV in SSA countries and has narrowed existing gaps in the literature. However, there is a need for further studies, including qualitative studies, to focus on interventions to reduce GBV.

## 5. Conclusions and Recommendation

Predictors that increase experiences of GBV include women and men with lower levels of education, increased levels of alcohol consumption by the woman and/or her partner, adolescent and young mothers, women with limited decision-making skills, women with a previous history of child abuse, women who have experienced depression, individual and community tolerant attitudes to GBV, employed women, low socioeconomic status and limited food security. Due to the complexity of factors associated with GBV, the need to develop an integrated comprehensive intervention approach is a top priority to eliminate GBV and reach the United Nation SDG targets of 2030 in SSA. This would include introducing socio-behavioural change communication interventions at both individual and community levels. Primary preventative methods that will address GBV in SSA should incorporate interventions that target both child and family abuse, increase access to educational facilities and result in women’s empowerment in their struggle towards elimination of all forms of violence.

## Figures and Tables

**Table 1:** Summarised description of studies on the risk of GBV.

Author	Country	Sample Size	Study Design	Identified Risk Factors for GBV
Bleck et al. (2015) [14]	Multi-country	44,487	Cross-sectional	The average initiation of sex abuse occurs after union 3.4 (3.4, 3.5) in SSA
Yaya et al. (2019) [15]	Angola	7669	Cross-sectional	Alcohol drinking by partners was associated with significantly higher odds of experiencing physical [OR: 2.95; 95% CI (2.63, 3.30)], emotional [OR: 2.5; 95% CI (2.2, 2.8)], and sexual IPV [OR: 2.73; 95% CI (2.22, 3.35)] among women. Women who reported experiencing physical IPV had increased odds of drinking alcohol [OR: 1.47; 95% CI (1.29, 1.68)] compared with those who did not. Place of residence—rural/urban: physical [OR: 0.74; 95% CI (0.63, 0.88)], emotional [OR: 0.71; 95% CI (0.59, 0.85)]. Education level—female: Complete primary/none: physical [OR: 0.71; 95% CI (0.55, 0.93)], emotional [OR: 0.76; 95% CI (0.57, 1.02)], sexual [OR: 0.72: 95% CI (0.44, 1.18)] and any type of violence [OR: 0.68; 95% CI (0.53, 0.88)]; Complete secondary/none: physical [OR: 0.74; 95% CI (0.61, 0.90)], emotional [OR: 0.96; 95% CI (0.779, 1.186)], sexual [OR: 1.07; 95% CI (0.76, 1.50)] and any type of violence [OR: 0.78; 95% CI (0.64, 0.94)]; Secondary/none: physical [OR; 0.56; 95% CI (0.40, 0.76)], emotional [OR: 0.80; 95% CI (0.58, 1.11)], sexual [OR: 0.61; 95% CI (0.32, 1.14)], and any type of violence [OR: 0.62; 95% CI (0.46, 0.83)]; Higher/none: physical [OR: 0.65; 95% CI (0.42, 1.01)], emotional[OR: 0.97; 95% CI (0.62, 1.52)], sexual [OR: 0.86; 95% CI (0.37, 1.99)], and any type of violence [OR: 0.78; 95% CI (0.52, 1.18)]. Education level—husband: Complete primary/none: physical [OR: 1.33; 95% CI (1.11, 1.60)], emotional [OR: 1.45; 95% CI (1.19, 1.77)], sexual [OR: 1.01; 95% CI (0.79, 1.53)] and any type of violence [OR: 1.49; 95% CI (1.25, 1.77)]; Complete secondary/none: only significant with any type of violence [OR: 1.25; 95% CI (1.04, 1.52)]; Secondary/none: not significant association; Higher/none: not significant association. Household head’s sex (Male): female/male: physical [OR: 0.83; 95% CI (0.72, 0.96)], emotional [OR: 0.86; 95% CI (0.74, 1.00)], and sexual IPV [OR: 0.91; 95% CI (0.71, 1.17)], and any type of violence [OR: 0.85; 95% CI (0.74, 0.97)]
Green et al. (2017) [16]	SSA-14	86,024	Cross-sectional	Partner alcohol use was associated with a 3.2-fold increase in the odds of IPV [OR: 3.2; 95% CI (2.94, 3.48)]. Partner alcohol use was associated with a significant increase in the odds of reporting IPV for all 14 countries included in this analysis.
Fawole et al. (2013) [17]	Nigeria	323	Cross-sectional	Beggars with higher knowledge levels [AOR: 0.23; 95% CI (0.07, 0.80)] and with more egalitarian attitudes [AOR: 0.38; 95% CI (0.12, 0.91)] were less likely to experience violence. Among homemakers, the predictors were younger age [AOR: 0.89; 95% CI (0.37, 0.79)], being widowed [AOR: 0.07; 95% CI (0.06, 0.74)], and having poor (traditional) attitudes to gender issues and justifying VAW [AOR: 0.36; 95% CI (0.14, 0.74)]
Ezeanchi et al. (2011) [18]	Nigeria	9614	Cross-sectional	Women with tolerant attitudes were more likely to have reported spousal physical (OR = 0.07, *p*, 0.001), sexual (OR = 0.15, *p*, 0.001) and emotional (OR = 0.06, *p* = 0.001) abuse. However, women with husbands with tolerant attitudes towards IPV were more likely to have reported spousal physical abuse (OR = 0.05, *p* = 0.034). At the community level, having an increasing number of women with tolerant attitudes towards IPV was positively associated with spousal sexual (OR = 1.39, *p* = 0.010) and emotional abuse (OR = 0.60, *p* = 0.007). Similarly, having an increasing number of women who had witnessed IPVAW in the community was positively associated only with spousal physical abuse (OR = 1.38, *p* = 0.004). There was positive correlation between all three types of IPVAW at both the individual level (physical vs. emotional [OR = 3.75, *p*,0.001]; physical vs. sexual [OR = 4.07, *p*, 0.001]; and sexual vs. emotional [OR = 4.31, *p*, 0.001]) and the community level (physical vs. emotional [OR = 0.48, *p* = 0.001]; physical vs. sexual [OR = 0.65, *p* = 0.014]; and sexual vs. emotional [OR = 0.57, *p* = 0.008]).
Mootz et al. (2018) [19]	Uganda	605	Cross-sectional	The indirect effect of armed conflict on IPV through the influence of partner alcohol use was significant for the respondents (*n* = 180) who made their own healthcare decisions [r: 0.10; 95% CI (0.02, 0.27)], as well as for women (*n* = 228) whose partner made their healthcare decisions [r: 0.25; 95% CI (0.12, 0.46)], but not for women (*n* = 150) who made household decisions jointly with their partners [r: 0.04; 95% CI (0.10, 0.19)].
Gibbs et al. (2018) [20]	South Africa	680	Cross-sectional	Women who had a partner, but did not live with them, were less likely to experience IPV (AOR: 0.53, *p* < 0.05), compared to women who lived with their partners. Household food insecurity was associated with more IPV experience, whereby those reporting the highest levels of food insecurity were more likely to report IPV experience, compared to those reporting no or little food insecurity (AOR: 1.84; *p* < 0.02). IPV experience was associated with more controlling behaviours (AOR: 1.14, *p* < 0.0001), and more quarrelling in the relationship (AOR: 1.57, *p* < 0.0001). Women reporting IPV experience reported more alcohol use (AOR: 1.06, *p* < 0.002) and drug use (compared to none) (AOR: 1.75, *p* < 0.0025). Women reporting IPV was associated with higher depression scores (AOR: 1.02, *p* < 0.034).
Adjah et al. (2016) [21]	Ghana	1524	Cross-sectional	Risk factors for domestic violence (Place of residence—urban/rural [OR: 1.35; 95% CI (1.08, 1.70)]; Educational level—partner: higher/no education [OR: 0.05; 95% CI (0.34, 0.8)]; husband drinks alcohol—Yes/No [OR: 2.52; 95% CI (2.04, 3.12)] and respondents’ mother beat father—Yes/No [OR: 3.04; 95% CI (1.61, 5.76)]; respondents’ father beat mother—Yes/No [OR: 1.41; 95% CI (1.02, 1.96)].
Admasu et al. (2016) [22]	Ethiopia	300	Cross-sectional	In multivariable logistic regression, three variables, i.e., dowry payment impact [OR: 8.7; 95% CI (4.2, 17.9)], partner education and undergoing marriage ceremony—No/Yes [OR: 4.1; 95% CI (2–8.2)] were associated. When compared with literate partners, illiterate partners were 50% less likely to use violence against their intimate partner during recent pregnancy [AOR: 0.5; 95% CI (0.2, 0.9)].
Fawole et al.(2018) [23]	Nigeria	640	Cross-sectional	Females were less likely to experience physical violence [AOR: 0.3; 95% CI (0.2–0.4)] and psychological violence [AOR: 0.6; 95% CI (0.4, 0.8)]. Students who were in a relationship and who had a history of parental violence were more likely to experience sexual [AOR: 1.7; 95% CI (1.2, 2.4)] and [AOR: 1.5; 95% CI (1.2, 2.2)] and psychological [AOR: 1.3; 95% CI (1.1, 1.5)] and [AOR: 1.3] violence.
Okenwa et al. (2009) [24]	Nigeria	934	Cross-sectional	Demographic variables such as age (15–24 ref) 25–34 years [OR: 0.33; 95% CI (0.09, 1.21)], 35–44 years [OR: 0.096; 95% CI (0.043–0.692)], 45–49 years [OR: 0.362; 95% CI (0.047–2.807)], and having children [OR: 0.56; 95% CI (0.31, 0.99)] remained significantly associated with IPV after adjusting for possible confounding with other independent study variables. Contribution to household expenses of more than half [OR: 3.84; 95% CI (1.57, 9.39)] as compared to no contribution, i.e., women’s autonomy and contribution to household expenses independently predicted IPV.
Berhane et al.(2015) [25]	Ethiopia	422	Cross-sectional	Age at first marriage greater than or equal to 17 years [AOR: 4.42; 95% CI (2.07, 9.42)], women with no formal education [AOR: 2.78; 95% CI (1.10, 7.08)], rural dwellers [AOR: 2.63; 95% CI (1.24, 5.58)], intimate partners with no formal education [AOR: 2.78; 95% CI (1.10, 7.08)] and intimate partner alcohol consumption [AOR: 3.8; 95% CI (1.85, 7.82)] were factors associated with intimate partner physical violence towards pregnant women.
Gust et al. (2017) [26]	Kenya	7421	Cross-sectional	Five factors were associated with physical violence by a sexual partner among women: being married or cohabiting [OR: 2.04; 95% CI (1.60, 2.59)], low education [OR: 1.16; 95% CI (1.03, 1.30)], and reporting forced sex in the last 12 months [OR: 2.53; 95% CI (2.23, 2.88)], partner alcohol/drug use [OR: 1.82; 95% CI (1.61, 2.08)], and deliberately terminating a pregnancy—yes/no [OR: 1.45; 95% CI (1.21, 1.74)].
Kimani et al. (2016) [27]	Kenya	301	Cross-sectional	Factors that showed a significant association with vulnerability to sexual violence against adolescent girls were alcohol use [OR: 1.82; 95% CI (1.61, 2.08)], and family connectedness [OR: 10.6; *p* < 0.001)].
Fute et al. (2015) [28]	Ethiopia	660	Cross-sectional	Female sex [AOR: 2.00; 95% CI (1.28, 2.39)], short work experience [AOR: 8.86; 95% CI (3.47, 22.64)], age group of 22–25 [AOR: 4.17; 95% CI (2.46, 7.08)], age group of 26–35 [AOR: 1.9; 95% CI (1.16, 3.1)], working in the emergency department [(AOR: 4.28; 95% CI (1.39, 4.34)] and working in the inpatient department [AOR: 2.11; 95% CI (1.98, 2.64)] were associated with violence.
Zegenahegn et al. (2019) [29]	Uganda	908	Cross-sectional	Decision-making explanatory variable—men’s perspective: Jointly: large household purchase [r = −0.059 (*p* < 0.030)], husband’s earnings [r = −0.091, *p* < 0.035]; Wife alone: large household purchase [r = −0.117, *p* < 0.041], husband’s earnings [r = −0.158, *p* < 0.053]
Fesehan et al. (2012) [30]	Ethiopia	422	Cross-sectional	Significant risk factors associated with experiencing physical violence were being a farmer [AOR: 3.0; 95% CI (1.7, 5.5)], knowing women in the neighbourhood whose husbands beat them [AOR: 1.87; 95% CI (1.0, 3.5)], being a Muslim [AOR: 2.4; 95% CI (1.107, 5.5)] and having a drunkard partner [AOR: 2.1; 95% CI (1.0, 4.5)].
Bui et al. (2019) [31]	Zimbabwe	5280	Cross-sectional	Husband having more education: physical violence—27.11%; sexual—14%; and emotional—24.35%. Compared with women who have the same level of education as their husbands or partners, women whose husbands have a higher level of education than theirs have higher reporting physical violence [OR: 1.4, *p* < 0.01] and a 32% higher likelihood of reporting emotional violence [OR: 1.32, *p* < 0.05]; women who have a higher level of education than their husbands have a 66% higher likelihood of reporting sexual violence [OR: 1.66, *p* < 0.01] and 41% higher likelihood of reporting emotional violence [OR: 1.41, *p* < 0.05]. In addition, living in rural areas and longer marital duration also increase the likelihood of physical and sexual violence. Among women living in rural areas, the likelihood of reporting physical violence is 31% higher (OR: 1.31, *p* < 0.01), and the likelihood of reporting sexual violence is 24% higher (OR: 1.24, *p* < 0.05) than that among women living in urban areas. Women who have lived with their husband for 5 years or longer also have 48% higher likelihood of reporting physical violence (OR: 1.48, *p* < 0.01) and 34% higher likelihood of reporting sexual violence (OR: 1.34, *p* < 0.05) than those who have lived with their husband less than 5 years. The likelihood of reporting physical violence is 23% higher among women living with their husbands than among those who did not live in the same household as their husbands (OR: 1.23, *p* < 0.05).
Pack et al. (2014) [32]	Kenya	619	Cross-sectional	Supporting one to two other people [OR: 2.14; 95% CI (1.07, 4.27)], experiencing child abuse [OR: 2.47; 95% CI (1.54, 3.95)], witnessing mother abuse [OR: 1.64; 95% CI (1.05, 2.57)], and greater alcohol consumption [OR: 2.60; 95% CI (1.56–4.32)] were risk factors for IPV in this sample.
Matseke et al. (2017) [33]	South Africa	673	Cross-sectional	Levels of depressive symptoms and greater perceived stigma were associated with combined physical and psychological IPV. Psychological IPV and physical IPV were also individually associated with greater perceived stigma and higher levels of depressive symptoms
Azene et al. (2019) [34]	Ethiopia	409	Cross-sectional	Lower educational status of partners [AOR: 3.26; 95% CI (1.45, 7.36)], rural residency [AOR: 4.04; 95% CI (1.17, 13.93)], frequent alcohol abuse by partner [AOR: 4.79; 95% CI (2.08, 11.04)], early initiation of antenatal care [AOR: 0.44; 95% CI (0.24, 0.81], age of women between 17 and 26 years [AOR: 0.21; 95% CI (0.09, 0.49)], and choice of partner by the woman only (AOR: 3.26; 95% CI (1.24–8.57)] were statistically significant factors associated with intimate partner violence towards pregnant women.
Pengpid et al. (2016) [35]	SSA-7(22)	16,979	Cross-sectional	Among both men and women, sociodemographic factors (first, second, third, or fourth year senior study [OR: 1.80; 95% CI (1.23, 2.64); 1.36 (0.93–2.00); 1.80 (1.25–2.60)], living in a low or lower middle income country) and risk factors (history of childhood physical and sexual abuse [OR: 2.37; 95% CI (1.56, 3.62)], [OR: 3.87; 95% CI (2.12, 7.06)], made someone pregnant or had been pregnant [OR: 1.87; 95% CI (1.38–2.53)], having had two or more sexual partners in the past 12 months [OR: 1.78; 95% CI (1.39, 2.29)], current tobacco use [OR: 1.30; 95% CI (1.01, 1.68)] and having PTSD symptoms [OR: 1.36; 95% CI (0.96, 1.94)] [OR: 1.63; 95% CI (1.21, 2.20)]) were associated with physical and/or sexual violence victimization.
Fawole et al. (2010) [36]	Nigeria	820	Cross-sectional	Predictors of perpetrating violence were being in polygamous unions [OR: 1.83, 95% CI (1.11, 3.03)], consuming alcohol [AOR: 1.67; 95% CI (1.10, 2.53)], and being Muslim [AOR: 1.87; 95% CI (1.21, 2.910)]. Men with inadequate knowledge and negative attitudes had greater likelihood of perpetrating IPV [AOR: 2.11; 95% CI (1.37, 3.26)] and IPV [AOR: 2.09; 95% CI (1.33, 3.27)]. IPV was also associated with young age.
Wandera et al. (2015) [37]	Uganda	1307	Cross-sectional	The odds of reporting IPSV were higher among women whose partners were jealous if they talked with other men [OR: 1.81; 95% CI (1.22, 2.68)], if their partners accused them of unfaithfulness [OR: 1.50; 95% CI (1.03, 2.19)] and if their partners did not permit them to meet with female friends [OR: 1.63; 95% CI (1.11, 2.39)]. The odds of IPSV were also higher among women whose partners tried to limit contact with their family [OR: 1.73; 95% CI (1.11, 2.67)] and often got drunk [OR: 1.80; 95% CI (1.15, 2.81)]. Finally, women who were sometimes or often afraid of their partners ([OR: 1.78; 95% CI (1.21, 2.60)] and [OR: 1.56; 95% CI (1.04, 2.40)], respectively) were more likely to report IPSV.
Hatcher et al. (2019) [38]	South Africa	2006	Cross-sectional	Food insecurity was associated with doubled odds of intimate partner violence [(OR: 2.15; 95% CI (1.73, 2.66)].
Lyons et al. (2017) [39]	Côte d’Ivoire	466	Cross-sectional	Police refusal of protection was associated with physical [AOR: 2.8; 95% CI (1.7, 4.4)] and sexual violence [(AOR: 3.0; 95% CI: 1.9, 4.8)]. Blackmail was associated with physical [(AOR: 2.5; 95% CI (1.5, 4.2)] and sexual violence [AOR: 2.4; 95% CI (1.5, 4.0)]. Physical violence was associated with fear [(AOR: 2.2; 95% CI (1.3, 3.1)] and avoidance of seeking health services [AOR: 2.3; 95% CI: 1.5, 3.8].
Stockl et al. (2010) [40]	Tanzania	1205	Cross-sectional	Women’s odds of drinking during their last pregnancy were significantly increased if they had experienced violence during pregnancy (Dar es Salaam: adjusted [OR: 5.63; 95% CI (2.97, 10.9)], *p* < 0.001; Mbeya: [OR: 3.78; 95% CI (2.43, 5.89), *p* < 0.001)]. Violence during pregnancy was also associated with having had a child or infant that died (Dar es Salaam: adjusted [OR: 1.89; 95% CI (1.14, 3.12), *p* < 0.05]; Mbeya: adjusted [OR: 1.73; 95% CI (1.12, 2.68), *p* < 0.05]. Women in Mbeya who had no formal education were significantly more likely to experience violence during pregnancy than women with primary education [OR: 1.87; 95% CI (1.24, 2.82), *p* < 0.01]. Similarly, such violence was more likely if their partner’s education level was lower [OR: 1. 76; 95% CI (1.14, 2.72), *p* < 0.05].
Mahenge et al. (2016) [41]	Tanzania	500	Cross-sectional	Physical and/or sexual IPV during pregnancy was associated with cohabiting [AOR: 2.2; 95% CI (1.24, 4.03)] and having a partner who was 25 years old or younger [AOR: 2.7, 95% CI (1.08–6.71)]. Postpartum, physical and/or sexual IPV was associated with having a partner who was 25 years old or younger [AOR: 4.4, 95% CI (1.24–15.6)]
Selin et al. (2019) [42]	South Africa	2533	Cohort	The prevalence of any IPV ever among AGYW aged 13 years to 14 years, 15 years to 16 years, and 17 years to 20 years was 10.8%, 17.7%, and 32.1%, respectively.
Ezeanochie et al. (2010) [18]	Nigeria	305	Cross-sectional	Identified risk factors for experiencing violence were multiparty (OR: 9.4; 95% CI (1.23, 71.33)], respondents with an HIV-positive child [OR: 9.2; 95% CI (4.53, 18.84)], experience of violence before they were diagnosed HIV positive (OR: 44.4; 95% CI (10.33, 190.42)] and women with partners without postsecondary education (OR: 2.3; 95% CI (1.40, 3.91)].
Delamou et al. (2015) [43]	Guinea	232	Cross-sectional	The odds of experiencing IPV was higher in women with secondary or vocational level of education than those with a higher level of education (AOR: 8.4; 95% CI: 1.2, 58.5). Women residing in other communes of Conakry (AOR: 5.6; 95% CI: 1.4–22.9) and those preferring injectable FP methods (AOR: 4.5; 95% CI (1.2–16.8)] were more likely to experience lifetime IPV.
Prabhu et al. (2011) [44]	Tanzania	2436	Cross-sectional	Adjusting for sociodemographics, [OR: 0.51; 95% CI (1.10, 2.07)] for married women and [OR: 2.25; 95% CI (1.63–3.10)] for divorced women, compared with single women.
Gibbs et al. (2017) [45]	South Africa	375	Cross-sectional	Women reporting more power in their sexual relationship had less IPV-victimisation [AOR: 0.22, 95% CI (0.09, 0.56), *p* < 0.001)], while higher depressive symptoms were associated with more IPV [AOR: 1.26, 95% (1.10, 1.44), *p* < 0.001)].
Decker et al. (2014) [46]	Nigeria and South Africa	453	Cross-sectional	IPV was associated with past-month alcohol consumption [AOR: 3.42; 95% CI (2.05, 5.69)], past-month binge drinking [AOR: 7.66; 95% CI (4.36, 13.47)], lifetime marijuana use [AOR: 4.66, 95% CI (2.21, 9.85)], condom non-use in last sexual encounter [AOR: 4.50; 95% CI (2.17, 9.34)], multiple past-year sex partners [AOR: 6.01; 95% CI (3.41, 10.60)], pregnancy [AOR: 1.70; 95% CI (1.03, 2.80)], transactional sex [AOR: 23.32; 95% CI (18.96, 28.69)], depressive symptoms [AOR: 3.05; 95% CI (2.10, 4.44)], suicidal ideation [AOR: 2.64; 95% CI (1.85, 3.76)], and poor self-rated health [AOR: 6.19; 95% CI (3.01, 12.74)].
Malan et al. (2018) [47]	Western Cape	150	Cross-sectional	The adjusted model predicting physical IPV found women who were at risk for depression were more likely to experience physical IPV [ORs: 4.42; 95% CI (1.88–10.41)], and the model predicting sexual IPV found that women who reported experiencing community violence were more likely to report 12-month sexual IPV [OR: 3.85, 95% CI (1.14, 13.08)].
Fawole et al. (2014) [6]	Nigeria	305	Cross-sectional	Sexual violence was significantly more experienced [AOR: 2.23; 95% CI (1.15–4.36)] by older female sex workers (FSWs) than their younger counterparts, by permanent brothel residents [AOR: 2.08; 95% CI (1.22–3.55)] and among those who had been in the sex industry for more than five years [AOR: 2.01; 95% CI (0.98, 4.10)]. Respondents with good knowledge levels of types of violence were less vulnerable to physical violence [AOR: 0.45; 95% CI (0.26–0.77)]. Psychological violence was more likely among FSWs who smoked [AOR: 2.16; 95% CI (1.26–3.81)]. Risk of economic violence decreased with educational levels ([AOR: 0.54; 95% CI (0.30, 0.99)] and [AOR: 0.42; 95% CI (0.22, 0.83)] for secondary and post-secondary, respectively).
Addo et al. (2018) [48]	Ghana	2000	Cross-sectional	Senior high school education or higher was protective of IPV [AOR: 0.51; 95% CI (0.30–0.86)]. Depression [AOR: 1.06 (1.04, 1.08)], disability [AOR: 2.30 (1.57; 3.35)], witnessing abuse of mother [AOR: 2.1.98 (1.44, 2.72)], experience of childhood sexual abuse [AOR: 1.46 (1.07–1.99)], having had multiple sexual partners in past year [AOR: 2.60; 95% CI (1.49–4.53)], control by male partner [AOR: 1.03; 95% CI (1.00–1.06)], male partner alcohol use in past year [AOR: 2.65, 95% CI (2.12, 3.31)] and male partner infidelity [AOR: 2.31; (1.72, 3.09)] were significantly associated with increased odds of past year physical or sexual IPV experience.
Chen et al. (2017) [49]	Tanzania	5371	Cross-sectional	Risk factors of past 12-month IPV perpetration included past 12-month IPV victimization, making cash or in-kind earnings had 3 times the odds of perpetrating violence against their partners compared with women who did not [AOR: 3.34; 95% CI [1.75, 6.37)], having autonomy in decision making for visits to the wife’s family, compared with partners who jointly decided, women who made most decisions had 3 times the odds of IPV perpetration (AOR: 3.22; 95% CI [1.24, 8.38]), and acceptance of justifications for wife beating [AOR: 2.53; 95% CI (1.24, 5.14)]. Women much younger than their partners had lower odds of IPV perpetration (women 5 to 9 years younger [AOR: 0.25; 95% CI (0.09, 0.64)] or 15+ years younger [AOR: 0.20; 95% CI (0.05, 0.77)] than their partners had reduced odds of perpetration, compared with participants the same age or older than their partners). Risk factors of past 12-month IPV victimization included past 12-month IPV perpetration [AOR: 7.45; 95% CI (2.59, 21.4)], educational attainment [AOR: 1.83; 95% CI (1.04, 3.22)], having children [AORs: 2.6; 95% CI (2.54, 2.70)], partner’s alcohol consumption; [AOR: 2.25; 95% CI (1.77, 2.88)], partner’s decision making, acceptance of justifications for wife beating, and exposure to parental IPV [AOR: 2.48; 95% CI (2.00, 3.07)]. Making cash or in-kind earnings was the only protective factor against victimization [AOR: 0.69; 95% CI (0.55, 0.87)].
Memiah et al. (2011) [50]	Kenya	3028	Cross-sectional	Factors associated with experiencing IPV included women who: belonged to religions other than Catholic [AOR: 2.4; 95% CI (1.2, 4.9)] or Protestant [OR: 2.3; 95% CI (1.2, 4.5)]; resided in urban areas [OR; 1.4; 95% CI (1.1, 1.9)]; were currently working—YES/NO [OR: 1.6; 95% CI (1.3, 2.1)]; had a poor Wealth Index [OR: 1.3, 95% CI (1, 1.7)]; were not sexually assertive (OR: 1.1 (0.6, 2)]; experienced an early sexual debut of less than 18 years; and felt that their partners were justified in beating them [OR: 1.7 95% CI (1.3, 2.2)].
Were et al. (2014) [51]	SSA-7	3408	Cohort study	Those who were HIV-infected were more likely to report IPV [AOR: 1.33, *p* < 0.05; for men [AOR: 2.20, *p* < 0.001)].
Falb et al. [52]	Côte d’Ivoire	981	Controlled trial	In the final adjusted analyses, lifetime IPV was associated with a 3.7 increase in odds of reporting reproductive coercion (95% CI: 2.4–5.8) compared to women who did not report such victimization.
Breiding et al. (2011) [53]	Swaziland	1244	Cross-sectional	Compared with respondents who had been close to their biological mothers as children, those who had not been close to them had higher odds of having experienced sexual violence [OR: 1.89; 95% CI (1.14, 3.14)], as did those who had had no relationship with them at all [OR: 1.93; 95% CI (1.34; 2.80)]. In addition, greater odds of childhood sexual violence were noted among respondents who were not attending school at the time of the survey [OR: 2.26; 95% CI (1.70; 3.01)]; who were emotionally abused as children [OR: 2.04; 95% CI (1.50, 2.79)]; and who knew of another child who had been sexually assaulted [OR: 1.77; 95% CI: 1.31, 2.40)] or was having sex with a teacher [OR: 2.07; 95% CI (1.59–2.69)].
Mutagoma et al. (2019) [54]	Uganda	1978	Cross-sectional	In multivariable analysis, being aged 25 years old and above [AOR: 2.1; 95% CI (1.80–2.39)] compared to being aged less than 25 years old, having a regular boyfriend [AOR: 1.6; 95% CI (1.17, 2.30)] compared to not having a regular boyfriend, and having STI symptoms in the last 30 days [AOR: 1.5; 95% CI (1.01, 2.14)] compared to those without STI symptoms were positively associated with sexual violence. In multivariable analysis, being aged 25 years and above [AOR: 0.8; 95% CI (0.76–0.89)] compared to being aged less than 25 years old, and not drinking alcohol every day [AOR: 0.6, 95% CI (0.42, 0.87)] compared to drinking alcohol everyday were associated with lower odds of physical violence.
Bamiwoy et al. (2014) [55]	Multicountry (6)	38,426	Cross-sectional	Both bivariate and multivariate analyses show that in two of the six countries—Zambia and Mozambique—experience of violence is significantly higher among women from non-poor (rich) households than those from other households (poor and middle). For Zimbabwe and Kenya, women from poor households are more likely to have experienced spousal violence before than those from non-poor households. In the remaining two countries—Nigeria and Cameroon—middle class women are more likely to have suffered abuse before from a husband/partner than those from poor and rich households.
Deyessa et al. (2010) [56]	Ethiopia	1994	Cross-sectional	Rural women aged 25–34/15–24: [OR: 1.3; 95% CI (1.0, 1.7)]; literate/illiterate rural women [OR: 1.2; 95% CI (1.0, 1.5)]; and urban poverty status extreme poverty/rich [OR: 3.5, 95% CI (1.5, 8.4)]; respondent alone—rural and literate/urban and literate [AOR: 2.2; 95% CI (1.3, 3.7)]; rural and illiterate/urban and literate [OR: 1.6; 95% CI (1.1, 2.6)]; spouse alone—urban and illiterate/urban and literate [OR: 1.5, 95% CI (1.1, 2.5)]; rural—both literate [OR: 1.2, 95% CI (1.2, 4.3)]; woman literate, husband illiterate [OR; 3.4; 95% CI (1.7 6.9)]; woman illiterate, husband literate [OR: 2.2; 95% CI (1.3, 3.7)]; both illiterate [OR: 1.8; 95% CI (1.1, 3.1)].
Yenealem et al. (2019) [57]	Ethiopia	531	Cross-sectional	Working at emergency departments [AOR: 3.99; 95% CI (1.49, 10.73)], working shifts [AOR: 1.98; 95% CI (1.28, 3.03)], short experiences [AOR: 3.09; 95% CI: (1.20, 7.98)], and being a nurse or midwife [AOR: 4.06; 95% CI (1.20, 13.74)] were positively associated with workplace violence. The main sources of violence were visitors/patient relatives followed by colleagues and patients.
Ahinkorah et al. (2018) [58]	SSA	84,486	Cross-sectional	The odds of reporting experiencing IPV before were higher among women with decision-making capacity [AOR: 1. 35; 95% CI (1.35–1.48)]. Women who belong to other religious groups and Christians were more likely to experience IPV compared to those who were Muslims ([AOR: 1.73; 95% CI (1.65, 1.82)] and [AOR: 1.87; 95% CI (1.72, 2.02)], respectively). Women who have partners with no education [AOR: 1.11; 95% CI (1.03, 1.20)], those whose partners had primary education [AOR: 1.34; 95% CI (1.25, 1.44)] and those whose partners had secondary education [AOR: 1.22; 95% CI (1.15, 1.30)] were more likely to experience IPV compared to those whose partners had higher education. The odds of experiencing IPV were high among women who were employed compared to those who were unemployed [AOR: 1.33; 95% CI (1.28, 1.37)]. The likelihood of the occurrence of IPV was also high among women who were cohabiting compared to those who were married [AOR: 1.16; 95% CI (1.10, 1.21)]. Women with no education [AOR: 1.37; 95% CI (1.24, 1.51)], those with primary education [AOR: 1.65; 95% CI (1.50, 1.82)] and those with secondary education [AOR: 1. 50; 95% CI (0.37–1.64)] were more likely to experience IPV compared to those with higher education. Finally, women with the poorest wealth status [AOR: 1.28; 95% CI (1.20, 1.37)], those with poorer wealth status [AOR: 1.24; 95% CI (1.17, 1.32)], those with middle wealth status [AOR: 1.27; 95% CI (1.20, 1.34)] and those with richer wealth status [AOR: 1.11; 95% CI (1.06, 1.17)] were more likely to IPV compared to women with the richest wealth status.
Sisawo et al. (2011) [59]	Gambia	219	Cross-sectional	The perpetrators were mostly patients’ escorts/relatives followed by patients themselves. Perceived reasons of workplace violence were mainly attributed to nurse–client disagreement, understaffing, shortage of drugs and supplies, security vacuum, and lack of management attention to workplace violence.
Newman et al. (2011) [60]	Rwanda	297	Cross-sectional	The study identified gender-related patterns of perpetration, victimization and reactions to violence. Negative stereotypes of women, discrimination based on pregnancy, maternity and family responsibilities and the “glass ceiling” affected female health workers’ experiences and career paths and contributed to a context of violence. Gender equality lowered the odds of health workers experiencing violence. Rwandan stakeholders used study results to formulate recommendations to address workplace violence gender discrimination through policy reform and programs.
Hendrickson et al. (2018) [61]	Tanzania	496	Cross-sectional	Intraregional and inter-regionally mobile FSWs had 1.9 times greater odds of reporting recent GBV [OR: 1.89; 95% CI (1.06, 3.38)] compared with non-mobile FSWs and a 2.5 times higher relative risk for recent experience of severe GBV relative to no recent GBV (OR: 2.51; 95% CI (1.33, 4.74)).

## Supplementary Materials

The following are available online at https://www.mdpi.com/article/10.3390/ijerph18094407/s1, Figure S1: Prisma flow chart that describes selection process of GBV articles that met inclusion criteria; Table S1. Appraisal of Quantitative Studies using CASP; Figure S2. Funnel plot for studies focused on education and GBV; Figure S3: Funnel plot for studies on alcohol consumption and GBV; Figure S4: Funnel plot for studies focused on substance use and GBV; Figure S5. Funnel plot for studies on decision-making skills and GBV; Figure S6. Funnel plot for studies on tolerant attitudes and GBV; Figure S7. Funnel plot for studies on history of child abuse and GBV; and Figure S8. Funnel plot for studies on family history of abuse and GBV.
