# Supplementary material for: A Systematic Review and Meta-Analysis of Associated Factors of Gender-Based Violence against Women in Sub-Saharan Africa
Source: Int J Environ Res Public Health. 2021 Apr 21;18(9):4407. doi: 10.3390/ijerph18094407 (PMC8122481; doi:10.3390/ijerph18094407)
Supplement: Supplementary file 1 [file ijerph-18-04407-s001.zip › ijerph-1125809-supplementary.pdf]

Table S1. Appraisal of Quantitative Studies

Authors	Question 1	Question 2	Question 3	Question 4	Question 5	Question 6	Question 7	Question 8	Question 9	Question 10	Quality of paper
Bleck et al. (2015)	Yes	Yes	Yes	Yes	Yes	Yes	Yes	Yes	NO	Clear	High
Yaya et al. (2019)	Yes	Yes	Yes	Yes	Yes	Yes	Yes	Yes	Yes	Clear	High
Greene, et al. (2017)	Yes	Yes	Yes	Yes	Yes	Yes	Yes	Yes	Yes	Clear	High
Fawole et al. (2013)	Yes	Yes	Yes	NO	Yes	Yes	Yes	Yes	Yes	Clear	High
Mootz et al. (2018)	Yes	Yes	Yes	Yes	Yes	Unclear	Yes		Yes	Clear	High
Decker et al.(2014)	Yes	Yes	Yes	Yes	Yes	NO	Yes	NO	Yes	Clear	Medium
Adjah et al. (2016)	Yes	NO	Yes	Yes	Yes	Yes	Yes	Yes	Yes	unclear	Medium
Admasu et al. (2016)	Yes	Unclear	Yes	NO	Yes	Yes	Yes	Yes	Yes	Clear	Medium
Fawole et al. (2018)	Yes	Unclear	Yes	NO	NO	Yes	Yes	NO	Yes	Clear	Medium
Okenwa et al. (2009)	Yes	Yes	Yes	Yes	Yes	Yes	Yes	Yes	Yes	Clear	High
Berhane et al. (2015)	Yes	Yes	Yes	Yes	Unclear	Yes	Yes	Yes	Yes	Clear	High
Gust et al. (2017)	Yes	Unclear	Yes	Yes	Yes	Yes	Yes	Yes	Yes	Clear	High
Kimani et al. (2016)	Yes	Yes	Yes	NO	Yes	Yes	Yes	Yes	Yes	Clear	High
Gibbs et al.(2017)	Yes	Unclear	Yes	Yes	Unclear	Yes	Yes	Yes	7	Clear	Medium
Fute et al. (2015)	Yes	Unclear	Yes	Yes	Unclear	Yes	Yes	Yes	Yes	Clear	Medium
Fesehan et al. (2012)	Yes	Unclear	Yes	Yes	Yes	Yes	Yes	Yes	Yes	Clear	High
Zegenahegn et al. (2019)	Yes	No	Yes	Yes	NO	Yes	Yes	Yes	Yes	Clear	Medium
Bui et al. (2016)	Yes	No	Unclear	Yes	Yes	Yes	Yes	Yes	Yes	Clear	Medium
Pack et al. (2013)	Yes	No	Unclear	Yes	NO	Yes	Yes	NO	Yes	Clear	Fair
Matsekeet al. (2017)	Yes	No	Unclear	Yes	Yes	Yes	Yes	Yes	Yes	Clear	Medium

Azene et al. (2019)	Yes	No	Yes	Unclear	Yes	Yes	Yes	Yes	Yes	Clear	Medium
Deyessa et al. (2009)	Yes	Unclear	Yes	Yes	Yes	Yes	Yes	Yes	Yes	Clear	High
Ahinkorah et al(2018)	Yes	Unclear	A	Yes	Yes	Yes	Yes	Yes	Yes	Clear	High
Pengpid et al. (2016)	Yes	Unclear	Yes	Yes	NO	Yes	Yes	Yes	Yes	Clear	Medium
Fawole et al. (2010)	Yes	Yes	NO	Yes	Yes	Yes	Yes	Yes	Yes	Clear	High
Sisawo et al.(2011)	Yes	Unclear	Yes	Yes	NO	Yes	Yes	Yes	Yes	Clear	Medium
Newman et al. (2011)	Yes	Yes	Yes	Yes	Yes	Yes	Yes	Yes	Yes	Clear	High
Wandera et al. (2015)	Yes	Unclear	Yes	Yes	Yes	Yes	Yes	Yes	Yes	Clear	High
Hatcher et al.(2019)	Yes	Yes	Unclear	Yes	Yes	Yes	Yes	Yes	Yes	Clear	high
Oumeora (2017)	Yes	NO	Yes	NO	Yes	Yes	Yes	Yes	Yes	Clear	Medium
Malan et.al(2018)	Yes	Yes	Yes	NO	Yes	Yes	Yes	Yes	Yes	unclear	Medium
Stockl et al. (2010)	Yes	Yes	NO	Yes	NO	Yes	Yes	NO	Yes	Clear	Fair
Mahenge et al. (2016)	Yes	Unclear	Yes	Yes	NO	Yes	Yes	Yes	Yes	Clear	Medium
Selin et al. (2019)	Yes	Yes	Yes	Yes	Yes	Yes	Yes	Yes	Yes	Clear	high
Ezeanochie, et al. (2010)	Yes	NO	Yes	NO	Yes	Yes	Yes	NO	Yes	Clear	Medium
Prabhu, et al. (2011)	Yes	NO	Yes	Yes	Yes	Yes	Yes	Yes	Yes	Clear	High
Fawole et al. (2014)	Yes	Yes	Yes	NO	Yes	Yes	Yes	Yes	Yes	Clear	High
Addo et al(2017)	Yes	Unclear	Yes	Yes	Yes	Yes	Yes	Yes	Yes	Clear	High
Chen et al. (2017)	Yes	Yes	Yes	Yes	Yes	Yes	Yes	Yes	Yes	Clear	High
Memiah et al. (2018)	Yes	NO	Yes	Yes	Yes	Yes	Yes		Yes	Clear	High
Were et al. (2014)	Yes	NO	Yes	Yes	NO	Yes	Yes	NO	Yes	Clear	Medium
Falb, et al. (2014)	Yes	Yes	Yes	Yes	Yes	Yes	Yes	Yes	Yes	Clear	High
Mutagom et al. (2019)	Yes	Yes	Yes	Yes	Yes	Yes	Yes	Yes	Yes	Clear	High
Bamiwoy et al. (2014)	Yes	Yes	Yes	Yes	Yes	Yes	Yes	Yes	Yes	Clear	High
Yenealem et al. (2019)	Yes	Yes	Yes	Yes	Yes	Yes	Yes	Yes	Yes	Clear	High

Hendricks et al. (2018)	Yes	Yes	Yes	Yes	Yes	Yes	Yes	Yes	Yes	Clear	High

CASP Key Questions

1. Did the study address a clearly focused issue?
2. Was the participants of the study recruited in an acceptable way?
3. Was the outcome accurately measured to minimise bias?
4. Was the sampling appropriate for the study
5. What are the results of the study?
6. How precise the tools used to measure the results?
7. Do you believe the results?
8. Can the results be applied to the local population?
9. Do the results of the study fit with other available evidence?
10. What are the implications of this study for practice?

Supplementary files: Meta-analysis: Test of Publication Bias using Funnel Plot and Eggers Test

A. Education focused studies

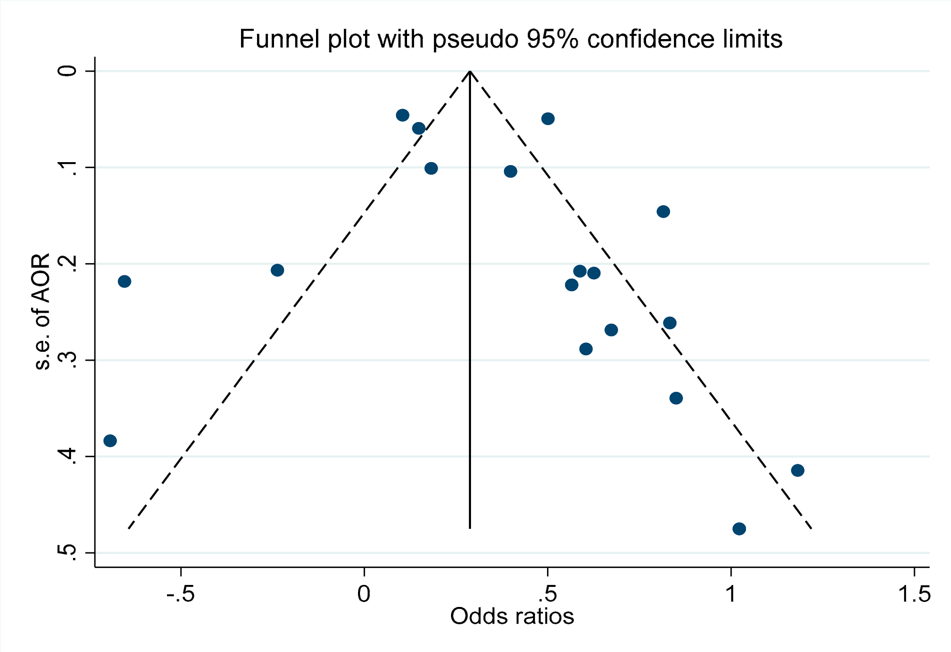

Figure S2. Funnel plot for studies focused on education and GBV

Tests for Publication Bias for partner and women education

Education	n	Egger's	
		Bias	P
Partner	8	0.97	0.492
Women	11	0.92	0.434
overall	19	0.94	0.273

B. Alcohol consumption focused studies

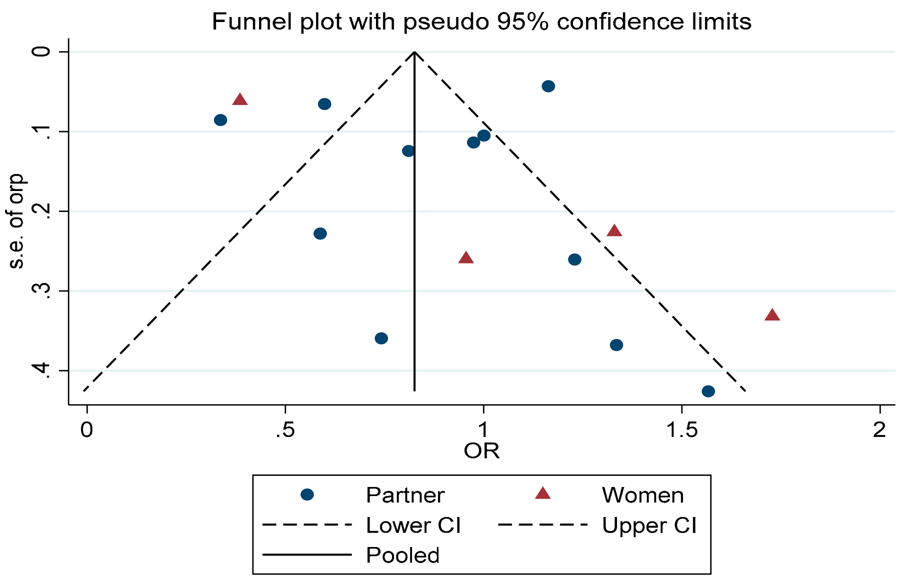

Figure S3: Funnel plot for studies on alcohol consumption and GBV

Tests for Publication bias for partner and women alcohol consumption

	n	Egger's	
Education		Bias	P
Partner	11	0.65	0.71
Women	4	4.5	0.038

C. Substance use focused studies

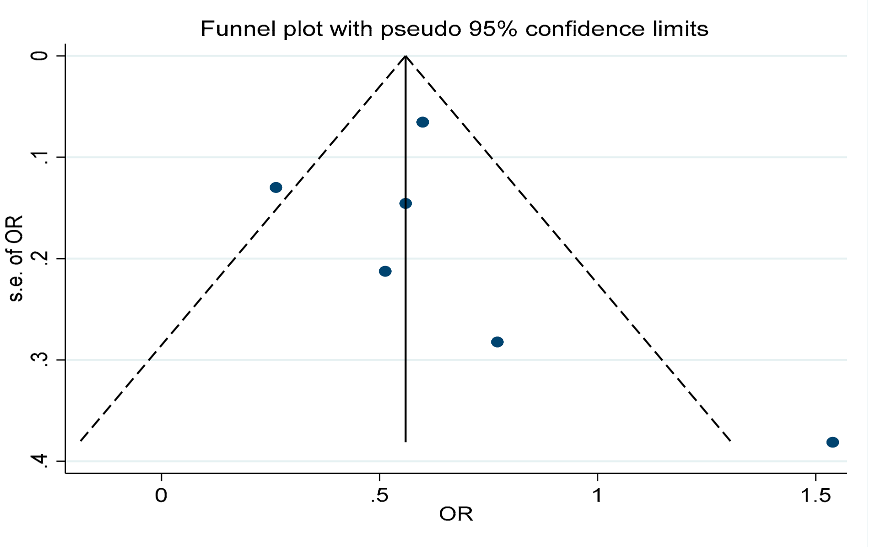

Figure S4: Funnel plot for studies focused on substance use and GBV

D. Decision- making skills focused studies

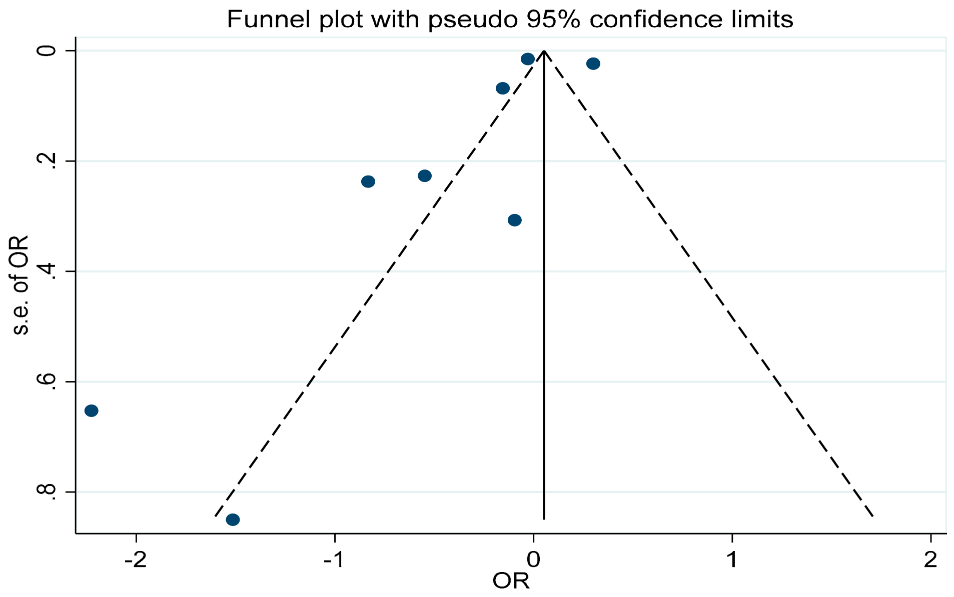

Figure S5. Funnel plot for studies on decision-making skills and GBV

E. Tolerant attitude focused studies

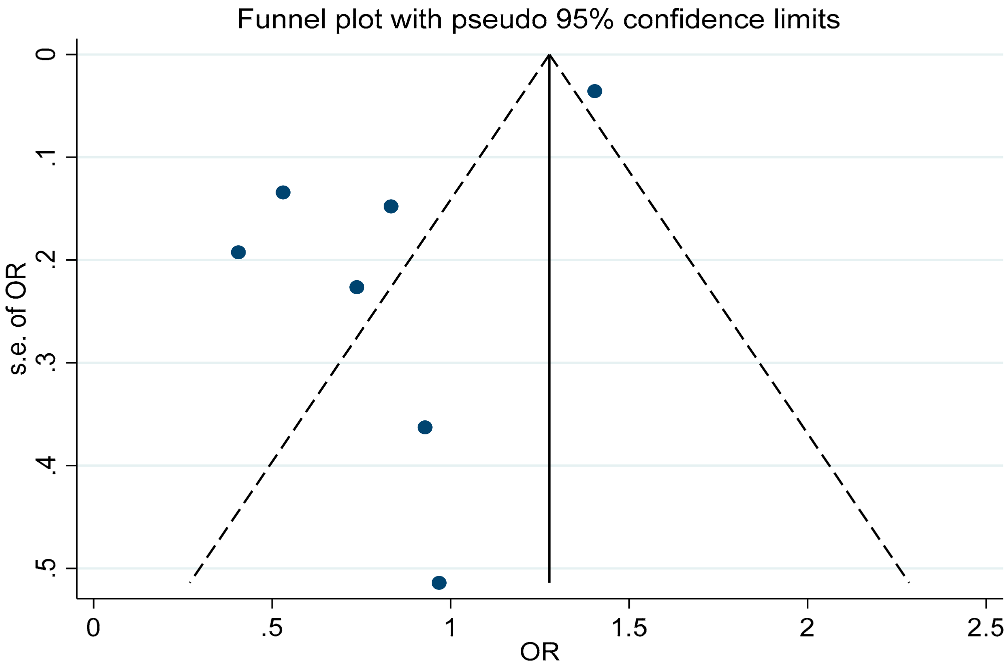

Figure S6. Funnel plot for studies on tolerant attitudes and GBV

F. History of child abuse focused studies

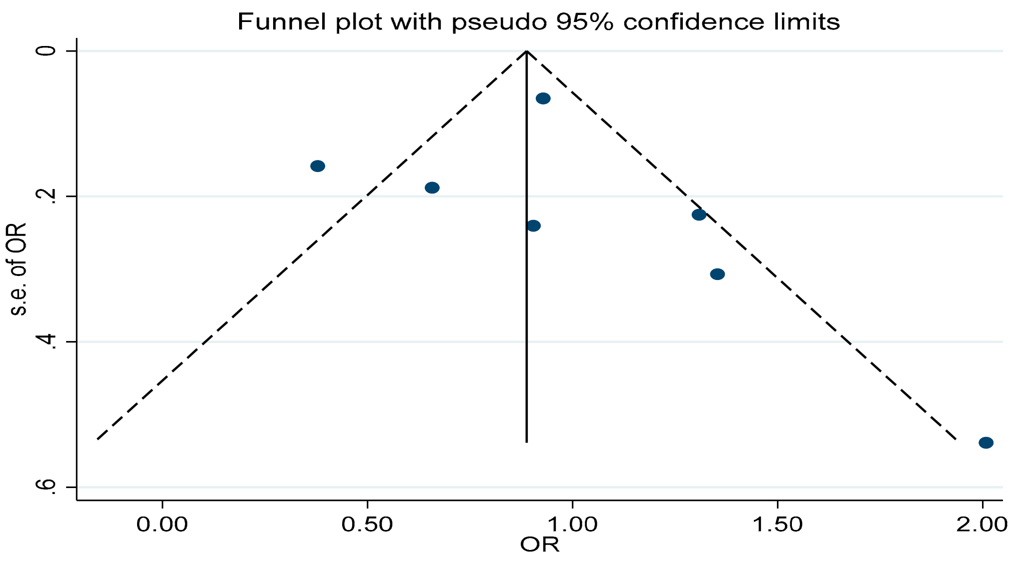

Figure S7. Funnel plot for studies on history of child abuse and GBV

G. History of beating focused studies

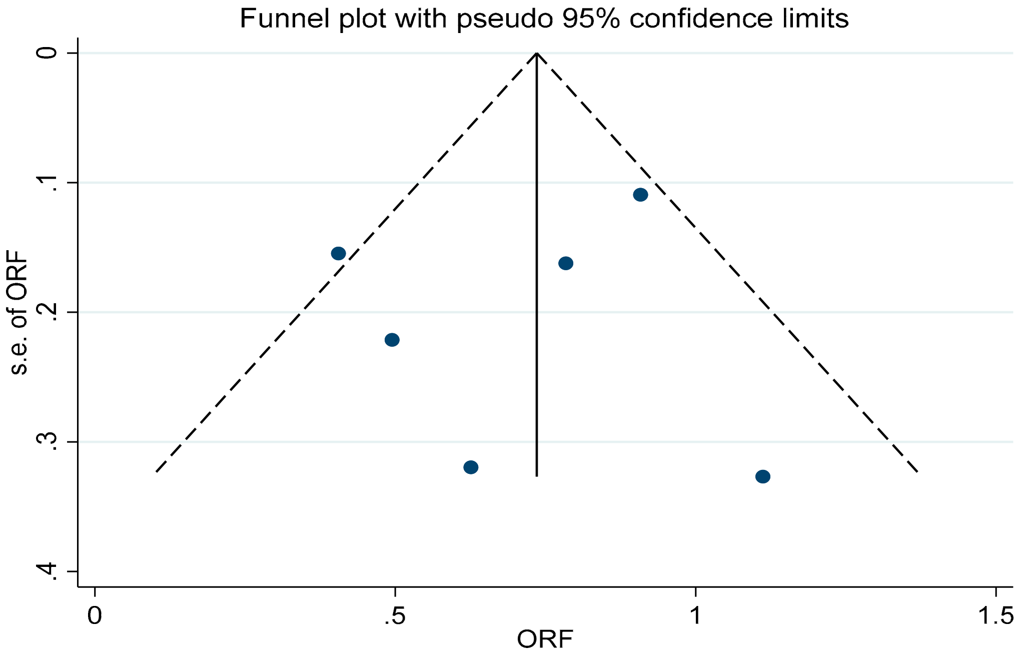

Figure S8. Funnel plot for studies on family history of abuse and GBV
